# A deep convolutional neural network approach to single-particle recognition in cryo-electron microscopy

**DOI:** 10.1186/s12859-017-1757-y

**Published:** 2017-07-21

**Authors:** Yanan Zhu, Qi Ouyang, Youdong Mao

**Affiliations:** 1 Center for Quantitative Biology, Peking University, Beijing, 100871 China; 2 State Key Laboratory for Artificial Microstructure and Mesoscopic Physics, Peking University, Institute of Condensed Matter Physics, School of Physics, Beijing, 100871 China; 3 Peking-Tsinghua Center for Life Sciences, Peking University, Beijing, 100871 China; 4 Intel Parallel Computing Center for Structural Biology, Department of Microbiology and Immunobiology, Dana-Farber Cancer Institute, Harvard Medical School, Boston, MA 02115 USA

**Keywords:** Cryo-EM, Particle recognition, Convolutional neural network, Deep learning, Single-particle reconstruction

## Abstract

**Background:**

Single-particle cryo-electron microscopy (cryo-EM) has become a mainstream tool for the structural determination of biological macromolecular complexes. However, high-resolution cryo-EM reconstruction often requires hundreds of thousands of single-particle images. Particle extraction from experimental micrographs thus can be laborious and presents a major practical bottleneck in cryo-EM structural determination. Existing computational methods for particle picking often use low-resolution templates for particle matching, making them susceptible to reference-dependent bias. It is critical to develop a highly efficient template-free method for the automatic recognition of particle images from cryo-EM micrographs.

**Results:**

We developed a deep learning-based algorithmic framework, DeepEM, for single-particle recognition from noisy cryo-EM micrographs, enabling automated particle picking, selection and verification in an integrated fashion. The kernel of DeepEM is built upon a convolutional neural network (CNN) composed of eight layers, which can be recursively trained to be highly “knowledgeable”. Our approach exhibits an improved performance and accuracy when tested on the standard KLH dataset. Application of DeepEM to several challenging experimental cryo-EM datasets demonstrated its ability to avoid the selection of un-wanted particles and non-particles even when true particles contain fewer features.

**Conclusions:**

The DeepEM methodology, derived from a deep CNN, allows automated particle extraction from raw cryo-EM micrographs in the absence of a template. It demonstrates an improved performance, objectivity and accuracy. Application of this novel method is expected to free the labor involved in single-particle verification, significantly improving the efficiency of cryo-EM data processing.

**Electronic supplementary material:**

The online version of this article (doi:10.1186/s12859-017-1757-y) contains supplementary material, which is available to authorized users.

## Background

Single-particle cryo-EM images suffer from heavy background noise and low contrast, due to the limited electron dose used in imaging in order to reduce radiation damage to the biomolecules of interest [1]. Hence, a large number of single-particle images, extracted from cryo-EM micrographs, is required to perform a reliable 3D reconstruction of the underlying structure. Particle recognition thus represents the first bottleneck in the practice of cryo-EM structure determination. During the past decades, many computational methods have been proposed for automated particle recognition, mostly based on template matching, edge detection, feature extraction or neural networks [2–15]. The template matching methods depend on a local cross-correlation that is sensitive to noise, and a substantial fraction of false positives may result from false correlation peaks [2–8]. Similarly, both the edge-based [9, 10] and feature-based methods [11–13] suffer from a dramatical reduction of performance with lower contrast of the micrographs. In a different approach, a method based on a three-layer pyramidal-type artificial neural network was developed [14, 15]. However, there is only one hidden layer in the designed neutral network, which is insufficient to extract rich features from single-particle images. A common problem for these automated particle recognition algorithms lies in the fact that they cannot distinguish “good particles” from “bad” ones, including overlapped particles, local aggregates, background noise fluctuations, ice contamination and carbon-rich areas. Thus, additional steps comprising unsupervised image classification or manual verification and selection are necessary to sort out “good particles” after initial automated particle picking. For example, TMaCS uses the support vector machine (SVM) algorithm to classify the particles initially picked by a template-matching method to remove false positives [16].

Deep learning is a type of machine learning that focuses on learning from multiple levels of feature representation, and can be used to make sense of multi-dimensional data such as images, sound and text [17–22]. It is a process of layered feature extraction. In other words, features in greater detail can be extracted by moving the hidden layer down to a deeper level using multiple non-linear transformations [22]. Convolutional neural network (CNN) is a biologically inspired deep, feed-forward neural network that has demonstrated an outstanding performance in speech recognition [23] and image processing, such as handwriting recognition [24], facial detection [25] and cellular image classification [26]. Its unique advantage lies in the fact that the special structure of shared local weights reduces the complexity of the network [27, 28]. Multidimensional images can be directly used as inputs of the network, which avoids the complexities of feature extraction in the reconstructed data [17, 27].

**Fig. 1:**
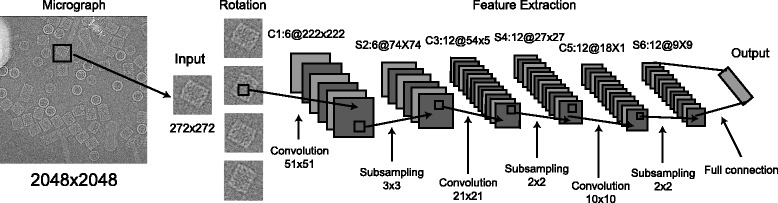
The architecture of the convolutional neural network used in DeepEM. The convolutional layer and the subsampling layer are abbreviated as C and S, respectively. C1:6@222×222 means that it is a convolutional layer and is the first layer of the network. This layer is comprised of six feature maps, each of which has a size of 222 × 222 pixels. The *symbols* and *numbers* above the feature maps of other layers have the equivalent corresponding meaning

The particle recognition problem in cryo-EM is fundamentally a binary classification problem, and is based on the features of single-particle images. We devised a novel automated particle recognition approach based on deep CNN learning [27]. Our algorithm, named DeepEM, is built upon an eight-layer CNN, including an input layer, three convolutional layers, three subsampling layers, and an output layer (Fig. 1). In this study, we applied this deep-learning approach to tackle the problem of automated template-free particle recognition. The DeepEM algorithm was examined through the task of detecting “good particles” from cryo-EM micrographs taken in a variety of situations, and demonstrated improved accuracy over other template-matching methods.

## Methods

### Design of the DeepEM algorithm

The DeepEM algorithm is based on a convolutional neural network, a multilayered neural network with local connections. It contains convolutional layers, subsampling layers and fully connected layers, in addition to the input and output layers (Fig. 1). The convolutional and subsampling layers produce feature maps through repeated application of the activation function across sub-regions of the images, which represent low-frequency features extracted from the previous layer (Additional file 1: Figure S1).

In the convolutional layer, which is the core building block of a CNN, the connections are local, but expand throughout the entire input image. Such a network architecture ensures that the outputs of the convolutional layer are effectively activated in response to the detection of meaningful input spatial features. The feature maps from the previous layer are convoluted by a learnable kernel. All convolution operation outputs are then transformed by a nonlinear activation function. We used the sigmoid function (1) as the nonlinear activation function.

1 $$ sigmoid(x)=1/\left(1+{e}^{- x}\right) $$

The convolution operations in the same convolutional layer share the same connectivity weights with the previous layer, so that:

2 $$ {X}_j^{\left[ l\right]}= sigmoid\left(\sum_{i\in {M}_j}{X_i^{\left[ l-1\right]}}^{\ast }{W}_{i j}^{\left[ l\right]}+{B}^{\left[ l\right]}\right), $$

where *l* represents the convolutional layer; *W* represents the shared weights; *M* represents different feature maps from the previous layer; *j* represents one of the output feature maps; *B* represents the bias in the layer; and the star symbol (*) represents the convolution operation.

Subsampling is another important concept in CNNs. A subsampling layer is designed to subsample the input data to progressively decrease the spatial size of the representation and reduce the number of parameters and computational cost in the network, thus reducing potential over-fitting [29]. We computed the subsampling averages after each convolutional layer using the following expression:

3 $$ {X}_{ij}^{\left[ l\right]}=\frac{1}{ M N}{\sum}_m^M{\sum}_n^N{X}_{iM+ m, jN+ n}^{\left[ l-1\right]} $$

where *i* and *j* represent the position of the output map; *M* and *N* represent the subsampling size in two orthogonal dimensions.

The basic network architecture of DeepEM contains three convolutional layers (the first, third, and fifth layers) and three subsampling layers (the second, fourth and sixth layers). The last layer is fully connected to the previous layer, which outputs a prediction for the classification of the input image by the weight matrix and the activation function (Fig. 1).

### Training of the DeepEM network

Prior to the application of DeepEM for automated particle recognition, the CNN needs to be trained with a manually assembled dataset, sampling both true particle images (positive training data) and non-particle images (negative training data) (Examples in Fig. 3a, b). Only a well-trained CNN should be used to recognize particles from raw micrographs. We used the error back-propagation method [30] to train the network, which produces an output of “1” for the true particle images and “0” for the non-particle images. The weights and biases in the CNN model are initialized with a random number between 0 and 1, and are then updated in the training process. We used the squared-error loss function [30] as the objective function in our model. For a training dataset with the number of *N*, it is defined as:

4 $$ {E}_N=\frac{1}{2 N}{\sum}_{n=1}^N{\left\Vert {t}_n-{y}_n\right\Vert}^2, $$

where *t*_*n*_ is the target of the *n*th training image, and *y*_*n*_ is the value of the output layer in response to the *n*th input training image. During the process of training, the objective function is minimized using an error back-propagation algorithm [30], which performs a gradient-based update as follows:

5 $$ \omega \left( t+1\right)=\omega (t)-\frac{\eta}{N}{\sum}_{k=1}^N{\varepsilon}_n\frac{\partial {\varepsilon}_n}{\partial \omega} $$

where *ε*_*n*_ = ‖*t*_*n*_ − *y*_*n*_‖; *ω*(*t*) and *ω*(*t* + 1) represent the parameters before and after the update of an iteration, respectively; *η* is the learning rate and was set to 1 in this study.

The data augmentation technique has shown a certain improvement in the accuracy of CNN training with a large number of parameters [14, 26]. During our DeepEM training, each original particle image in the training dataset was rotated by 90°, 180° and 270°, in order to augment the size of data sampling by a factor of four. The intensity of each pixel from an original or rotated image was then used as the input of a neuron of the input layer. The desired output was set to 1 for the positive data and 0 for the negative data in the error back-propagation procedure.

**Fig. 2:**
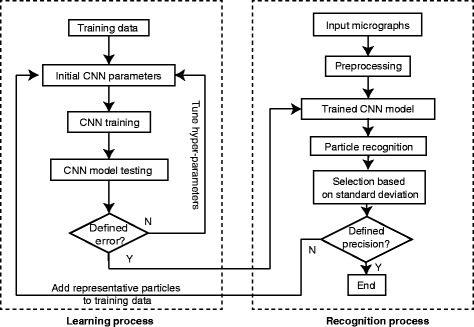
The workflow diagram of the DeepEM algorithm. The *dashed box on the left* represents the learning process; the *dashed box on the right* represents the recognition process

The experimental cryo-EM micrographs may contain heterogeneous objects, such as protein impurities, ice contamination, carbon-rich areas, overlapping particles and local aggregates. Moreover, since the molecules in the single-particle images assume random orientations, significantly different projection structures of the same macromolecule may coexist in a micrograph. These factors make it difficult to assemble a relatively balanced training dataset at the beginning, which must include representative positive and negative particle images. The initially trained CNN is prone to missing some target particles in certain views or recognizing some unwanted particles whose appearances are similar to the target. The training dataset can be optimized by adding a greater number of representative particle images to the original training dataset after testing on a separate set of micrographs that are independent of the ones used for assembling the original training dataset, and then re-training the network following the workflow chart shown in Fig. 2. After a sufficient number of iterations of training, the CNN becomes more “knowledgeable” in differentiating positive particles from negative ones.

Since the input particle images size may vary in different datasets, one can set different hyper-parameters for each case, including the number of feature maps, the kernel size of the convolutional layers and the pooling region size of the pooling layers. We empirically initialized these hyper-parameters and fine-tuned them during the training process (Fig. 2). The details of the hyper-parameters used in this study are shown in Table 1. In general, the output dimension of the convolutional layer is chosen as 70–90% of its input dimension, and the output dimension of the subsampling layer is scaled to about half its input dimension. We implemented the DeepEM algorithm based on the DeepLearnToolbox [31], a toolbox for the development of deep learning algorithms, in conjunction with Matlab.

**Table 1:** Hyper-parameters used in different datasets

Dataset	Particle size	Corresponding layer in DeepEM					
		C1	S2	C3	S4	C5	S6
KLH	272 × 272	6@222X222	6@74X74	12@54X54	12@27X27	12@18X18	12@9X9
19S	160 × 160	6@141X141	6@47X47	12@38X38	12@19X19	12@16X16	12@8X8
26S	150 × 150	6@120X120	6@60X60	12@46X46	12@23X23	12@14X14	12@7X7
Inflammasome	112 × 112	6@98X98	6@49X49	12@40X40	12@20X20	12@14X14	12@7X7

### Particle recognition and selection in the DeepEM model

When a well-trained CNN is used to recognize particles, a square box of pixels is taken as the CNN input. Each input image boxed out of a testing micrograph is rotated incrementally, to generate three additional copies of the input image with rotations of 90°, 180° and 270°, relative to the original. Each copy is used as a separate input to generate a CNN output. The final expectation value of each input image is taken as the average of its four output values from the non-rotated and rotated copies. The boxed area is initially placed into a corner of the testing micrograph, and is raster-scanned across the whole micrograph to generate an array of CNN outputs.

We used two criteria to select particles. First, a threshold score must be defined. The boxed image is identified as a candidate if the CNN output score of the particle is above the threshold score. Those particles whose CNN scores are below the threshold are rejected. We used the *F*-measure [32], which is a measure of the accuracy of a test that combines both precision and recall for binary classification problems, to determine the threshold score in our approach, which is defined as.

6 $$ {F}_{\beta}={\left(1+{\beta}^2\right)}^{\ast}\frac{precision^{\ast } recall}{\left({\beta^2}^{\ast } precision+ recall\right)}, $$

where *β* is a coefficient weighting the importance of precision and recall. In our method, we used the *F*_2_ score, which weights the recall higher than the precision. The *F*_2_-score reaches its best value at 1 and its worst at 0. We defined the cutoff threshold at the highest value of the *F*_2_-score.

Secondly, candidate images were further selected based on the standard deviation of the pixel intensities. There are often carbon-rich areas or contaminants in raw micrographs where the initially detected particles may not be good choices for downstream single-particle analysis. The pixels belonging to the “particles” in these areas usually have higher or lower standard deviations compared with those in other areas with clean amorphous ice. We therefore set a narrow range of the pixel standard deviation to remove the candidate particles that are initially picked from these unwanted areas [6, 16] (Additional file 1: Figure S2).

### DeepEM algorithm workflow

#### Learning process

**Input**: Training dataset.

**Output**: Trained CNN parameters (weights and biases)

1. Rotate each input particle image three times, each with a 90° increment;
2. Set the output of the positive data as 1, and the output of the negative data as 0;
3. Initialize the hyper-parameters;
4. Randomly initialize the weights and biases in each convolutional layer;
5. While (Learning error > Defined error), do
  - Tune the hyper-parameters or optimize the training dataset by adding more representative positive and negative particles from a new set of micrographs, which are independent of those used in the previous iterations, to the training dataset;
  - Train weights and biases via the error back-propagation algorithm;
  - Apply the trained CNN to an independent testing dataset to measure the learning error
6. End while

#### Recognition process

**Input**: Micrographs and trained CNN.

**Output**: Box files of selected particles in the EMAN2 [33] format for each micrograph

1. Iterate the following steps (a-c) until the whole micrograph has been raster-scanned;
  - Extract a square the size of a particle, starting from a corner of the input micrograph;
  - Rotate the boxed image three times, each with a 90-degree increment;
  - Use the trained CNN to process four copies of the boxed image, including the non-rotated and rotated copies, and average the resulting output scores of the four images;
2. Pick the particle candidates based on scores that are not only local maxima but also above the threshold score;
3. Select particle images based on their standard deviations;
4. Write the coordinates of the selected particle images into the box file.

### Performance evaluation

We evaluated the performance of the method based on the precision-recall curve [34], which is one of the most popular metrics for the performance evaluation of various particle-selection algorithms. The precision and recall are defined by Eqs. (7) and (8), respectively.

7 $$ \mathrm{Precision}=\frac{\mathrm{TP}}{\mathrm{TP}+\mathrm{FP}} $$

8 $$ \mathrm{Recall}=\frac{\mathrm{TP}}{\mathrm{TP}+\mathrm{FN}} $$

The precision represents the fraction of true positives (TP) among the total particle images selected (TP + FP), and the recall represents the fraction of true particle images selected among all the true particle images (TP + FN) contained in the micrographs. The precision-recall curve is generated from the algorithm by varying the threshold score used in the particle recognition procedure. When the threshold increases, the precision would increase and the recall would decrease accordingly. Thus, the threshold is manifested as a balance between the precision and the recall. For a good performance in particle selection, both the precision and the recall are expected to achieve higher values at a certain threshold.

### DeepEM training on the keyhole limpet Hemocyanin (KLH) dataset

**Fig. 3:**
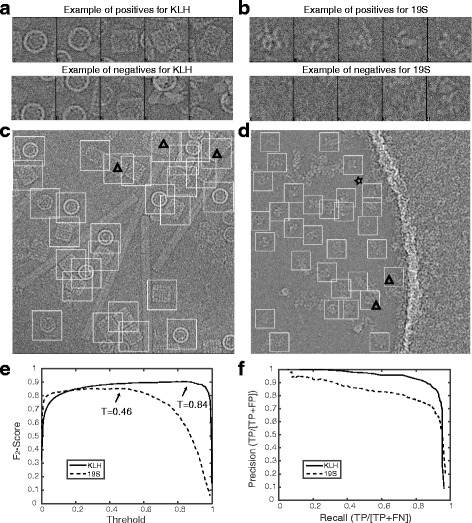
The DeepEM results for the KLH and 19S regulatory particle datasets. **a** and **b** Examples of positive and negative particle images selected for the CNN training in conjunction with the KLH and 19S datasets, respectively. **c** and **d** Typical micrographs from the KLH and 19S datasets, respectively. The *white square boxes* indicate the positive particle images selected by DeepEM. The *boxes with a triangle* inside indicate that a false-positive particle image was picked. The *star marks* one example of a false negative, a true particle missed by the recognition program. **e** The *F*_2_-score curves provide different thresholds for particle recognition in the KLH and 19S datasets, the arrows indicate the peaks of each curve, where the cutoff threshold value is defined. **f** The precision-recall curves plotted against a manually selected list of particle images

The KLH dataset was acquired from the US National Resource for Automated Molecular Microscopy (nramm.scripps.edu). KLH is ~8 MDa protein particle with a size of ~40 nm. It consists of 82 micrographs at 2.2 Å/pixel that were acquired on a Philips CM200 microscope at 120 kV. The size of the micrograph is 2048 by 2048 pixels. There are two main types of projection views of the KLH complex, the side view and the top view. We boxed the particle images with a dimension of 272 pixels. 800 particle images were manually selected for the positive training dataset. The same number of randomly selected non-particle images from the first fifty micrographs was used as a negative dataset (Fig. 3a). Each original image in the training dataset was rotated at 90° increments to create three additional images to augment the training data. We also selected some particle images as a testing dataset containing positive and negative data that were not used in the prior training step. The testing dataset was used to test the intermediately trained CNN model (Fig. 2). The accuracy or error of the CNN learning output from the testing dataset was used as a feedback parameter to tune the hyper-parameters, including the number of feature maps, kernel size of the convolutional layers, and subsampling size of the subsampling layers in the network. Throughout the training-testing cycles, we tuned the hyper-parameters and updated the training dataset until the accuracy of the CNN learning reached a satisfactory level. The acceptable value was often set as ~95% at the threshold of 0.5 (Fig. 2).

### Application to experimental cryo-EM data

The original sizes of the micrographs of the inflammasome, 19S regulatory particle and 26S proteasome were 7420 by 7676, 3710 by 3838 and 7420 by 7676 pixels, respectively. The pixel sizes of the inflammasome, 19S regulatory particle and proteasome holoenzyme were 0.86, 0.98 and 0.86 Å/pixel, respectively. For the inflammasome and 26S proteasome, the micrographs were binned 4 times. Therefore, the pixel size used for the inflammasome and proteasome holoenzyme was 3.44 Å/pixel. For the 19S regulatory particle, the micrographs were binned 2 times, resulting in a pixel size of 1.96 Å/pixel. Thus, the resulting sizes of the micrographs used in our tests were all 1855 by 1919 pixels; the dimension of the particle images of the inflammasome, 19S and 26S complexes were 112, 160 and 150 pixels, respectively. These experimental cryo-EM datasets were acquired using a FEI Tecnai Arctica microscope (FEI, USA) at 200 kV, equipped with a Gatan K2 Summit direct electron detector. Finally, we applied the DeepEM algorithm to these cryo-EM datasets. The hyper-parameters tuned for these datasets are shown in Table 1. Different from the training for the KLH dataset, we added true positive and false positive data, which were manually verified on a separate set of micrographs independent of the testing dataset used for tuning the hyper-parameters, to optimize the training dataset and to train the network recursively for the low-contrast datasets (Additional file 1: Figure S3).

## Results

### Experiments on the KLH dataset

We first tested our DeepEM algorithm on the Keyhole Limpet Hemocyanin (KLH) dataset [35] that was previously used as a standard testing dataset to benchmark various particle selection methods [3, 4, 6, 8, 11–13, 16]. For the KLH dataset, the recall and the precision both reached ~90% at the same time in the precision-recall curve (Fig. 3f) plotted against a manually selected set of particle images from 32 micrographs that did not include any particle images used in the training dataset. Our approach achieved a higher precision over all the particle images selected, whereas the recall was kept at a high value, indicating that fewer false-negative particle images were missed among the micrographs. In a typical KLH micrograph (Fig. 3c), all true particle images were automatically recognized by our method with a threshold of 0.84, as determined by the *F*_2_-score (see Methods and Eq. 6) (Fig. 3e). A comparison of the precision-recall curves between DeepEM, RELION [36] and TMACS [16] suggests that DeepEM outperforms these two template-matching based methods (Additional file 1: Figure S4).

**Fig. 4:**
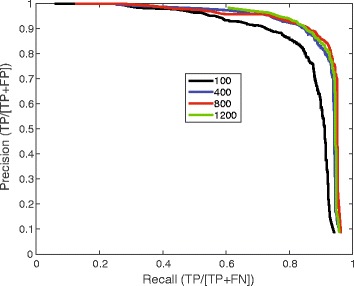
Impact of the training image number on the precision-recall curve. The *black*, *blue*, *red* and *green* curves were obtained with the training datasets including 100, 400, 800 and 1200 positive or negative images, respectively

To understand the impact of the number of training particles on algorithm performance, we varied the particle number in the KLH training dataset from 100 to 1200, and plotted the corresponding precision-recall curves (Fig. 4). In each testing case, the number of positive particles was kept equal to that of the negative particles. Although there was clear improvement in the precision-call curve when the training particle number was increased from 100 to 400, there was little improvement with a further increase of the training dataset size. The best result was obtained in the training run with 800 positive particle images.

### Experiments on cryo-EM datasets

We also applied our method to several challenging cryo-EM datasets collected using a direct electron detector, including the 19S regulatory particle, 26S proteasome and NLRC4/NAIP2 inflammasome [37]. Figure 3d shows a typical micrograph of the 19S regulatory particle, in which DeepEM selected almost all true particle images contained in the micrograph. At the same time, it avoided selecting non-particles from areas containing aggregates and carbon film. The precision-recall curve resulting from the test on the 19S dataset is shown in Fig. 3f. The precision and recall both reach ~80% at the same time. The picked particles were approximately as well-centered as the manually boxed ones. To further verify that the selected particle images are correct, we performed unsupervised 2D classification. The resulting reference-free class averages from about 100 micrographs were consistent with different views of the protein samples (Additional file 1: Figure S5).

**Fig. 5:**
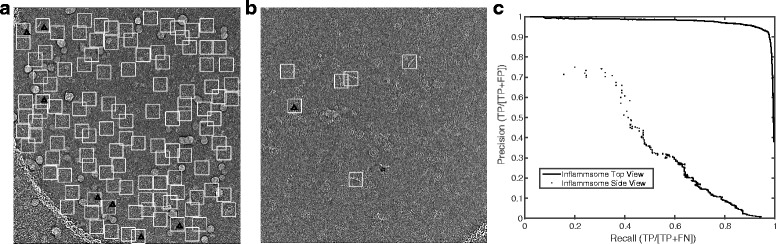
Two challenging examples of automated particle recognition. **a** A typical micrograph showing high-density top views of the inflammasome complex. Considerable ice contaminants and overlapping particles are present. **b** A typical micrograph of the side views of the inflammasome showing both a paucity of features and a low density of objects. The *white square boxes* indicate the positive particle images selected by DeepEM. The *boxes with a triangle* inside indicate that false-positive particle images were picked. The *boxes with a star* inside indicate the omitted particle images. **c** The precision-recall curves corresponding to the cases shown in (**a**) and (**b**)

Two difficult cases from the inflammasome dataset were examined. Figure 5a shows a micrograph with a high particle density that contains excessively overlapped particles and ice contamination. Most methods based on template matching were incapable of avoiding particle picking from overlapped particles and ice contaminants in this case. Figure 5b presents another difficult situation, in which the side views of the inflammasome display a lower SNR, lack low-frequency features, and are dispersed with a very low spatial density. In both cases, DeepEM still performed quite well in particle recognition, while avoiding the selection of overlapping particles and non-particles. Further tests on similar cases from other protein samples suggested that this observation had a good reproducibility (Additional file 1: Figure S6). Most importantly, DeepEM was able to determine the structure of the human 26S proteasome [38].

### Computational efficiency

The DeepEM algorithm was first tested on a Macintosh with a 3.3 GHz Intel Core i5 and 32 GB memory, running Matlab 2014b. When the size of the particle images increases, the parameter space increases substantially, so that it costs more computational time for each micrograph. We usually binned the original micrographs 2 or 4 times to reduce the size of the particle images. For the KLH dataset, it took about 7300 s per micrograph with a micrograph size of 2048 by 2048 pixels and particle image size of 272 by 272 pixels. For the 19S regulatory particle, inflammasome and 26S proteasome datasets, it took about 790, 560, and 1160 s per micrograph with a binned micrograph size of 1855 by 1919 pixels and particle image sizes of 112 by 112, 160 by 160, and 150 by 150 pixels, respectively. To speed up the calculations, multiple instances of the code were run in parallel. We also implemented a Graphic Processing Unit (GPU)-accelerated version of DeepEM in Matlab. We tested it on a desktop computer with 4.0 GHz Intel Core i7-6700 k, 64GB memory and Geforce GTX 970, running Matlab 2016a and CUDA 8.0. It only took about 190, 50, 40, and 60 s per micrograph for the KLH, 19S regulatory particle, inflammasome and 26S proteasome datasets, respectively. The GPU-accelerated DeepEM version therefore speeds up the computation by at least an order of magnitude.

## Discussion

Based on the principles of deep CNN, we have developed the DeepEM algorithm for single-particle recognition in cryo-EM. The method allows automated particle extraction from raw cryo-EM micrographs, thus improving the efficiency of cryo-EM data processing. In our current scheme, a new dataset containing particles of significantly different features may render the previously trained hyper-parameters suboptimal. Readers are directed to Table 1 as references for the hyper-parameter tuning for specific cases. Indeed, finding a set of fine-tuned hyper-parameters leading to optimized learning results on new datasets therefore demands additional user intervention in CNN training. In the above-described examples, we screened several combinations of hyper-parameters to empirically pinpoint an optimal setting. This procedure may be inefficient and can be laborious in certain cases. An automated method for the systemic tuning of hyper-parameters could be developed in the future to address this issue.

The execution of the DeepEM algorithm requires users to first label several hundreds of ‘good particles’ and ‘bad particles’ for CNN training purpose, which can be readily assembled from several micrographs. Further processing of these raw particle images is not needed. By contrast, in the traditional template-matching methods [2–8, 36], users need to first obtain many high-quality class averages or an initial 3D model, which involves multiple steps of single-particle analysis significantly more laborious than the single step of manual particle labeling required by our DeepEM approach. If the template is based on a 3D model, it is usually not trivial to determine a high-quality initial model from new samples, which involves a complete procedure of the ab initio 3D structure determination at low resolution [1]. If the template is based on a set of 2D class averages, users still have to first manually pick thousands of particles and then perform 2D image clustering to generate high-quality 2D classes. Moreover, the number of the reference images are often very limited and hardly include all kinds of orientations, potentially introducing orientation bias in particle picking through template matching. Thus, the preparation step of DeepEM is considerably easier than those of template-matching methods.

**Fig. 6:**
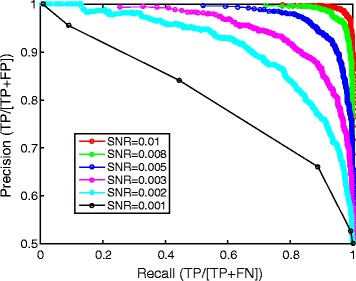
Effect of the signal-to-noise ratio (SNR) on the precision-recall curves. Three synthetic datasets were generated through computational simulation of micrographs containing single-particle images with SNRs of 0.01, 0.008, 0.005, 0.003, 0.002 and 0.001. For each case, the CNN was first trained on the synthetic dataset of a given SNR and then used to examine the precision-recall relationship using another synthetic dataset with the same SNR. All synthetic datasets used the 70S ribosome as the single-particle model

Although there are unlimited possibilities for the design of deep CNNs, we made some explorations that helped us understand the optimal use of CNNs for our single-particle recognition problem. First, we examined the noise tolerance of the algorithm with simulated datasets. When the SNR is decreased to 0.005, the DeepEM can still recognize particle images after proper training (Fig. 6). Second, we replaced the sigmoid activation function with a rectified linear unit (ReLU) function. Our results indicate that the ReLU function gives rise to a slightly inferior accuracy in particle recognition than the sigmoid function (Additional file 1: Figure S7). Third, we attempted to design a six-layer CNN, but found that it failed to produce a better or equivalent performance (data not shown). Thus, it is likely that the eight-layer CNN we designed possesses the minimum depth suited to our problem. A deeper CNN might enable greater capacities in these tasks and awaits further investigation. Finally, from the experiments on the inflammasome dataset, we noticed that DeepEM is more effective for feature-rich data. It exhibits a reduced performance when tested on the side views as compared to the top views of the inflammasome (Fig. 4c), because the side views exhibit significantly less low-frequency features than the top views. Thus, the richness of low-frequency particle features is positively correlated with the achievable performance of CNNs.

Our DeepEM algorithm framework exhibits several advantages. First, with sufficient training, DeepEM can select true particles without picking non-particles in a single, integrative step of particle recognition. In fact, it performs as well as a human worker. Similar performance was previously only made possible by combining several steps, encompassing automated particle picking, unsupervised classification and manual curation. Second, DeepEM features traits representative of other artificial intelligence (AI) or machine learning systems. The more it is trained or learned, the better it performs. We found that with iterative updating or optimization of the training dataset, the particle recognition performance of DeepEM can be further improved, which was not possible for conventional particle-recognition algorithms developed so far. Therefore, the performance of earlier algorithms was intricately bound by their mathematics and control parameters, and DeepEM overcomes these limitations.

## Conclusion

DeepEM, which is derived from deep CNNs, has proved to be a very useful tool for particle extraction from noisy micrographs in the absence of templates. This approach gives rise to improved “precision-recall” performance in particle recognition, and demonstrates a higher tolerance to much lower SNRs in the micrographs than was possible with older methods based on template-matching. Thus, it enables automated particle picking, selection and verification in an integrated fashion, with a quality comparable to that of a human worker. We expect that this development will broaden the applications of modern AI technology in expediting cryo-EM structure determination. Related AI technologies may be developed in the near future to address key challenges in this area, such as deep classification of highly heterogeneous cryo-EM datasets.

## Additional file

Additional file 1: Figure S1. The feature maps of the convolutional and subsampling layers from a typical particle image of KLH learned by our CNN. **Figure S2.** (a) and (b) show a comparison of the results obtained before and after additional selection using standard deviation of the KLH dataset, respectively. (c) and (d) show a comparison of the results obtained before and after additional selection using standard deviation of the 19S, respectively. **Figure S3.** (a) and (b) show a comparison of the results obtained before and after optimization of the training dataset, respectively. **Figure S4.** Comparison of DeepEM with TMACS and RELION using the KLH dataset as benchmark. The curves of TMACS [16] and RELION [36] were directly obtained from published data. **Figure S5.** Reference-free 2D classification of 19S proteasomes recognized by DeepEM. **Figure S6.** Results of the recognition of the side view of the 26S proteasome by DeepEM. **Figure S7.** A comparison of the results of different activation functions tested on the KLH dataset (PDF 477 kb)

## Abbreviations

AI: Artificial intelligence
CNN: Convolutional neural network
Cryo-EM: Cryo-electron microscopy
KLH: Keyhole Limpet Hemocyanin
SNR: Signal-to-noise ratio
