# Supplementary material for: A deep convolutional neural network approach to single-particle recognition in cryo-electron microscopy
Source: BMC Bioinformatics. 2017 Jul 21;18:348. doi: 10.1186/s12859-017-1757-y (PMC5521087; doi:10.1186/s12859-017-1757-y)
Supplement: Additional file 1: Figure S1. — The feature maps of the convolutional and subsampling layers from a typical particle image of KLH learned by our CNN. Figure S2. (a) and (b) show a comparison of the results obtained before and after additional selection using standard deviation of the KLH dataset, respectively. (c) and (d) show a comparison of the results obtained before and after additional selection using standard deviation of the 19S, respectively. Figure S3. (a) and (b) show a comparison of the results obtained before and after optimization of the training dataset, respectively. Figure S4. Comparison of DeepEM with TMACS and RELION using the KLH dataset as benchmark. The curves of TMACS [16] and RELION [36] were directly obtained from published data. Figure S5. Reference-free 2D classification of 19S proteasomes recognized by DeepEM. Figure S6. Results of the recognition of the side view of the 26S proteasome by DeepEM. Figure S7. A comparison of the results of different activation functions tested on the KLH dataset (PDF 477 kb) [file 12859_2017_1757_MOESM1_ESM.pdf]

Supplementary Information for

A deep convolutional neural network approach to single-particle recognition in cryo-electron microscopy

Yanan Zhu[1], Qi Ouyang[1,2,3], Youdong Mao[1,2,4]*

[1]Center for Quantitative Biology, [2]State Key Laboratory for Artificial Microstructure and Mesoscopic Physics, Institute of Condensed Matter Physics, School of Physics, [3]Peking-Tsinghua Center for Life Sciences at Academy for Advanced Interdisciplinary Studies, Peking University, Beijing 100871, China. [4]Intel Parallel Computing Center for Structural Biology, Dana-Farber Cancer Institute, Department of Microbiology and Immunobiology, Harvard Medical School, Boston, MA 02115, USA.

*Corresponding author. Tel: +1 617 632 4358. Fax: +1 617 632 4338. E-mail address: youdong_mao@dfci.harvard.edu (Y. M.).

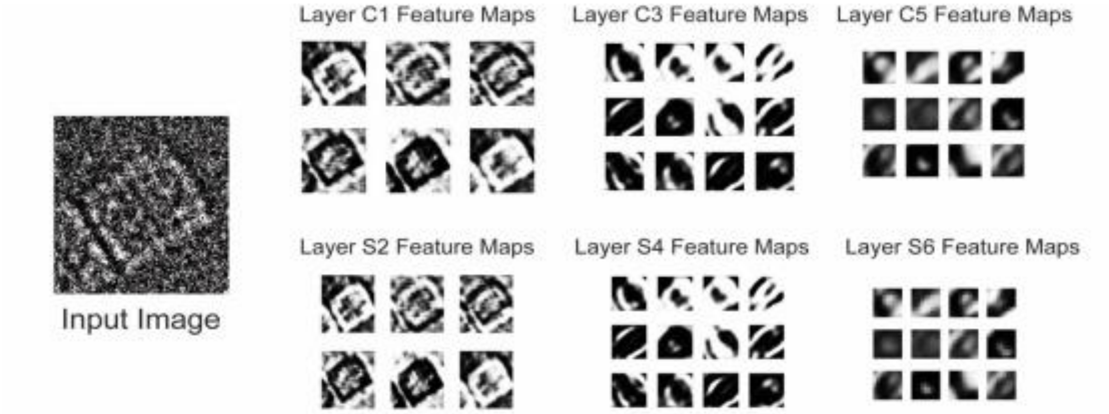

Supplementary Figure 1. The feature maps of convolutional and subsampling layers from a typical particle image of KLH learned by our CNN.

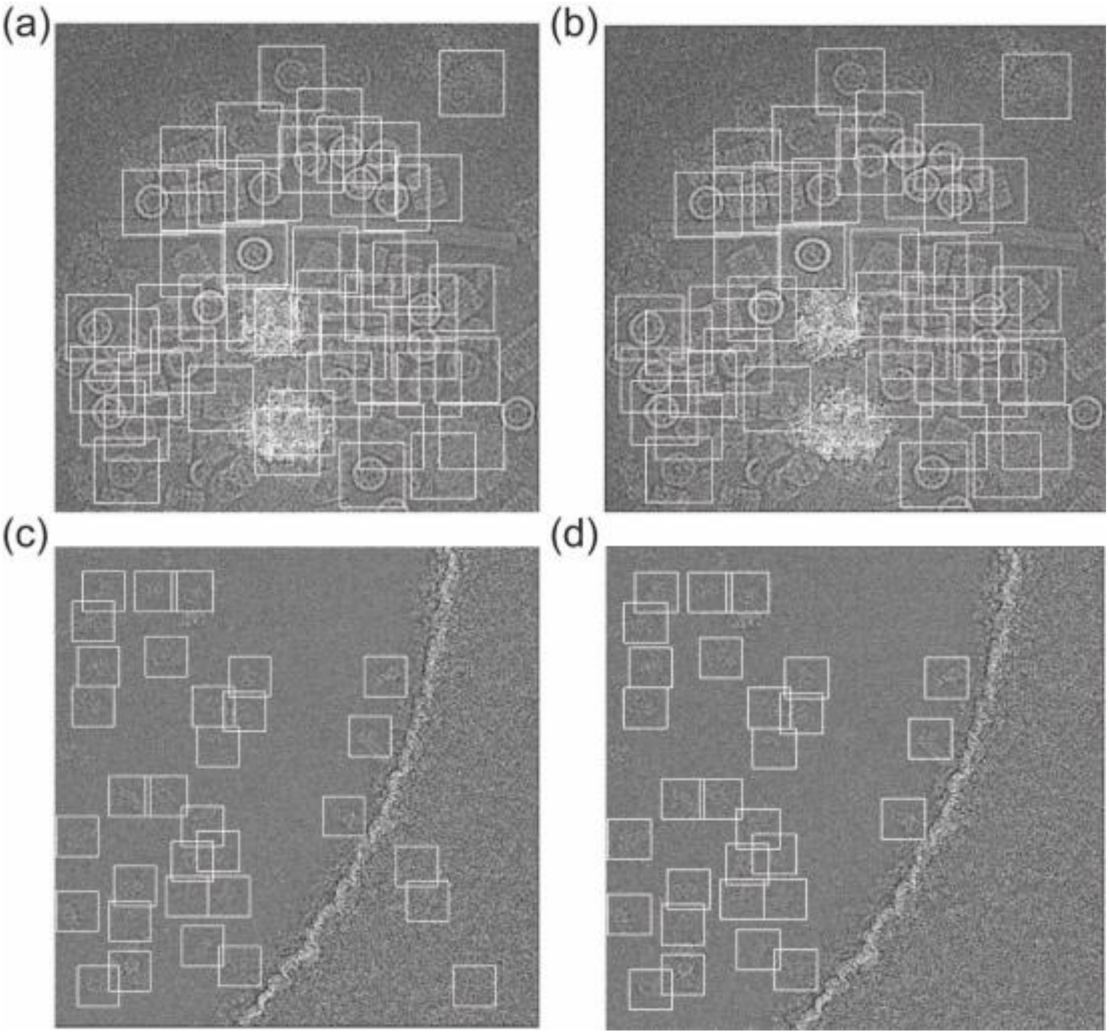

Supplementary Figure 2. (a) and (b) show the comparison of the results between before and after additional selection using standard deviation of the KLH dataset, respectively. (c) and (d) show the comparison of the results between before and after additional selection using standard deviation of the 19S, respectively.

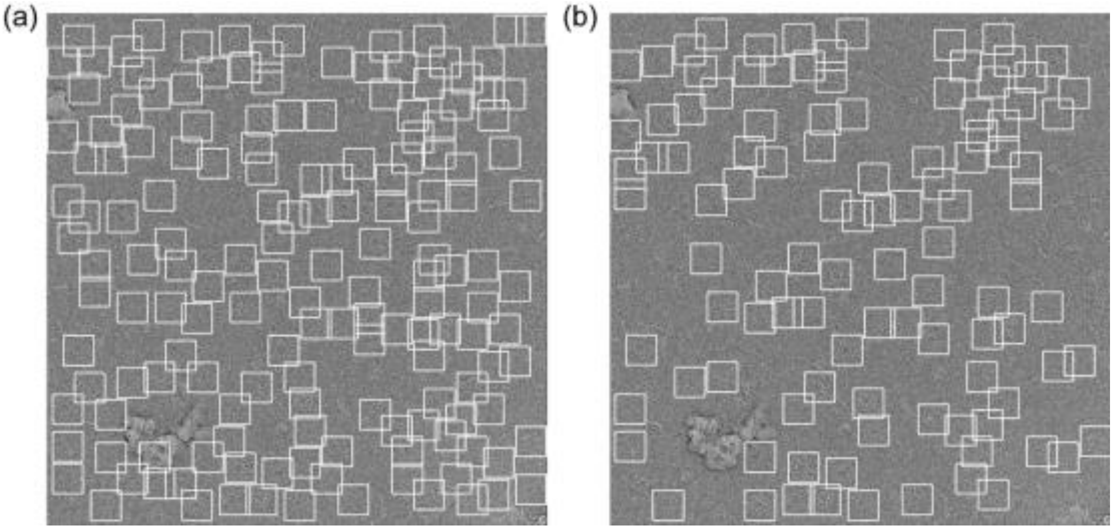

Supplementary Figure 3. (a) and (b) show the comparison of the results before and after optimization of the training dataset, respectively.

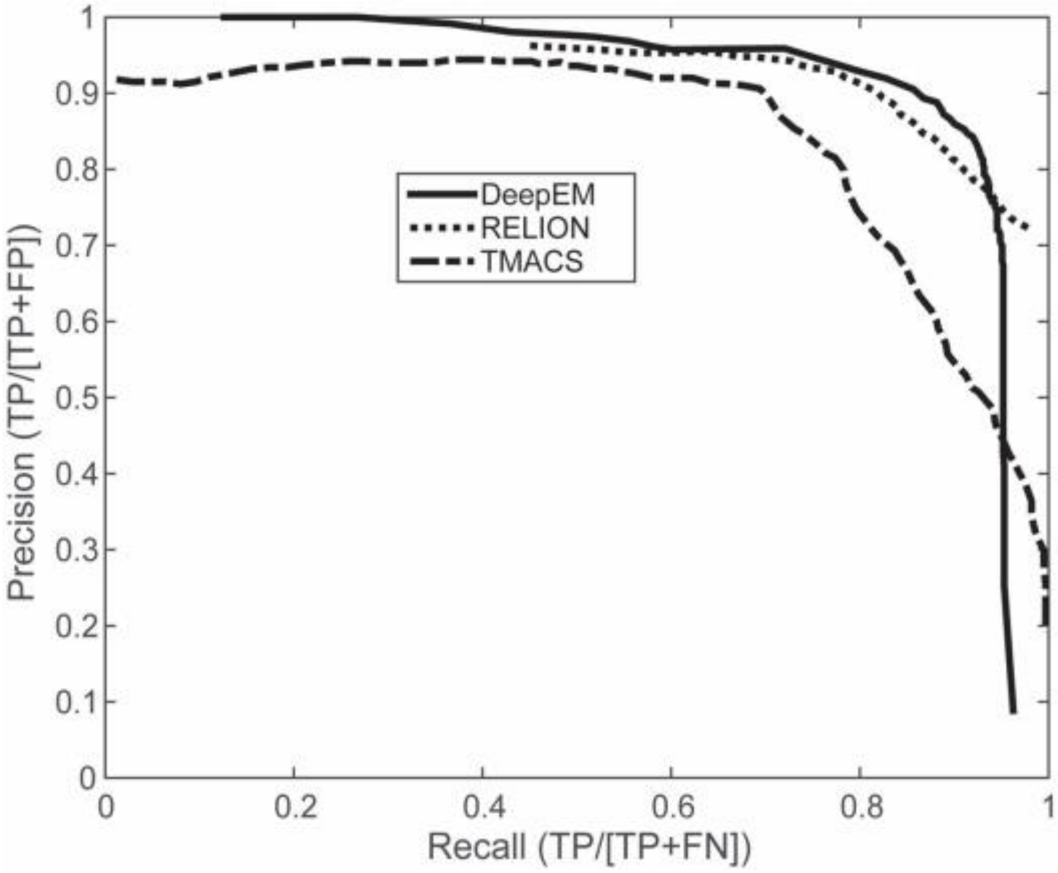

Supplementary Figure 4. Comparison of the DeepEM with TMACS and RELION in the KLH dataset. The curves of TMACS and RELION are obtained from the published data directly.

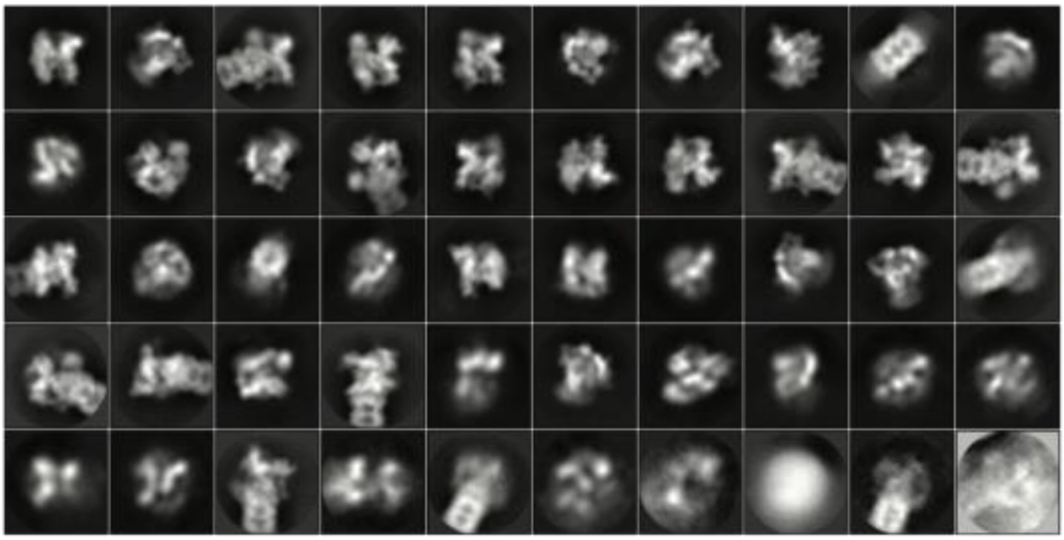

Supplementary Figure 5. The reference free 2D classification of 19S proteasomes recognized by DeepEM.

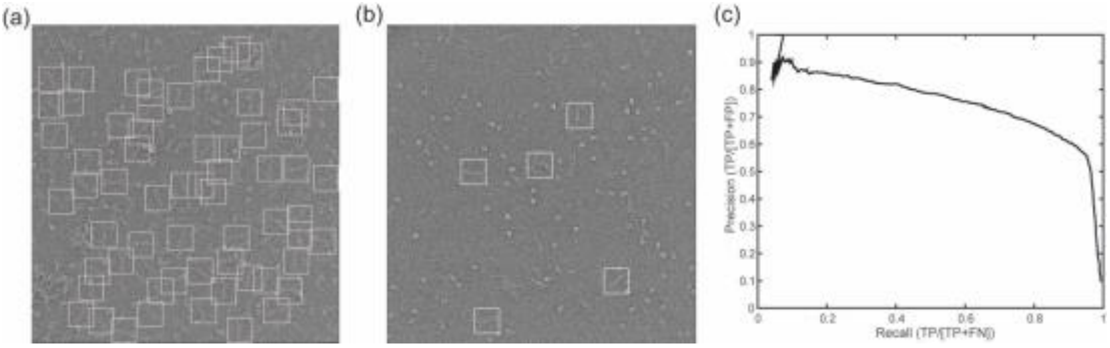

Supplementary Figure 6. The results in the recognition of the side view of 26S proteasome by DeepEM.

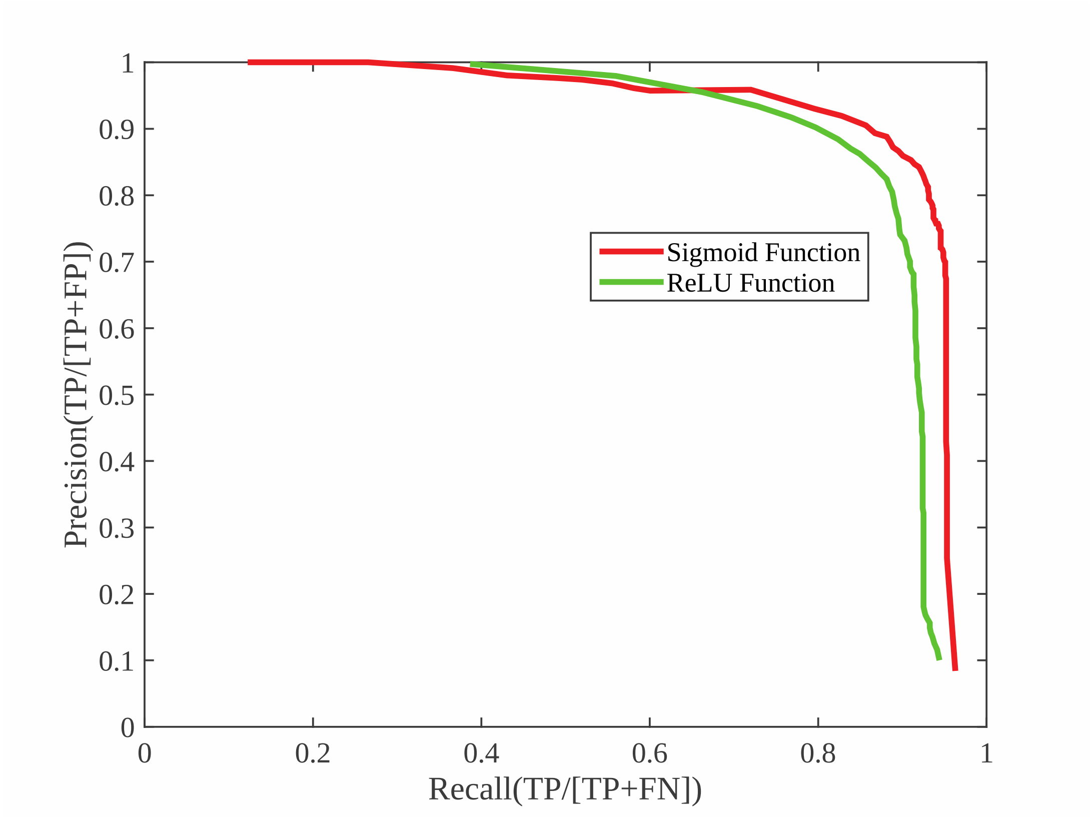

Supplementary Figure 7. The comparison results of different activation functions tested on the KLH dataset.
